# Unilateral Graves’ Disease With Ipsilateral Papillary Thyroid Cancer: A Case Report

**DOI:** 10.7759/cureus.80998

**Published:** 2025-03-22

**Authors:** Mohmmad H Alqattan, Mukhtar A Alqadhi, Ali M Alawadh, Mohammed A Almajed, Salman s Albakheet, Ali W Aldandan

**Affiliations:** 1 Radiology and Nuclear Medicine, Al-Ahsa Health Cluster, Al-Ahsa, SAU; 2 Pathology, Al-Ahsa Health Cluster, Al-Ahsa, SAU

**Keywords:** goiter, graves' disease, papillary thyroid carcinoma, ptc, unilateral graves' disease

## Abstract

Graves' disease, an autoimmune disorder characterized by diffuse goiter and hyperthyroidism, is commonly associated with bilateral thyroid involvement. However, unilateral Graves' disease is an extremely rare entity. Additionally, patients with Graves' disease have an increased risk of thyroid malignancy, particularly papillary thyroid carcinoma (PTC), which often presents with aggressive histological features. This case highlights the rare occurrence of unilateral Graves' disease with ipsilateral papillary thyroid carcinoma, emphasizing the importance of careful diagnostic assessment, including imaging and fine needle aspiration (FNA), in patients with atypical presentations. To the best of our knowledge, this is the third reported case of unilateral Graves' disease with ipsilateral thyroid malignancy and the first case documented in Saudi Arabia.

## Introduction

Graves' disease, also known as diffuse toxic goiter, is an idiopathic autoimmune disorder caused by elevated levels of autoantibodies directed against the thyroid-stimulating hormone (TSH) receptor (anti-TSH receptor antibodies) [1-2]. This leads to the diffuse enlargement of the thyroid gland and excessive production of thyroid hormones [1-2]. It is the most common cause of hyperthyroidism, with a higher prevalence in females than in males, and is frequently associated with thyroid ophthalmopathy [1].

Graves' disease typically affects both thyroid lobes; however, unilateral Graves' disease has been rarely reported in the literature [1,3-5]. It is well established that Graves' disease increases the risk of thyroid malignancy compared to the general population, and such malignancies often exhibit aggressive features [3,6-8]. Thyroid-stimulating hormone levels play a role in the development of thyroid cancer, as normal or elevated TSH levels are associated with an increased risk [9-10]. However, in Graves' disease, TSH levels are typically very low [1]. It is thought that the link between Graves’ disease and thyroid malignancy is due to anti-TSH receptor antibodies [3,11].

Furthermore, unilateral Graves' disease with ipsilateral thyroid malignancy is extremely rare [3,12]. To the best of our knowledge, our case represents the third reported instance in the literature and the first case documented in Saudi Arabia.

## Case presentation

The patient was a 43-year-old female who was a known case of hypertension and beta thalassemia trait with a prior surgical history of two cesarean sections, she presented with weight loss, palpitations, neck pain, and neck swelling worse on the right side for five to six months associated with dysphagia. There was no prior history of thyroid disease or neck irradiation. There was no history of chemotherapy administration. 

On physical examination, the thyroid gland revealed an enlarged right lobe, which was nontender and firm in consistency. The left thyroid lobe was normally palpable. There was no palpable cervical lymphadenopathy. The patient’s vital signs are tabulated in Table 1.

**Table 1:** The patient's vital signs on presentation

Vital signs	Value	References
Blood pressure	127/68 mmHg	Systolic <120 mmHg; diastolic <80 mmHg
Heart rate	74 beats/min	60-100 beats/min
Respiratory rate	20 breaths/min	12-18 breaths/min
Oxygen saturation	98% on room air	95%-100%

The patient's thyroid function laboratory workup is summarized in Table 2.

**Table 2:** The patient's thyroid function laboratory workup TSH: thyroid-stimulating hormone; free T3: tri-iodothyronine; free T4: thyroxine; L: low; H: high

Test	Result	Reference range
TSH	0.013 L	0.5-5.0 uIU/mL
Free T4	37.56 H	10.3-25.0 pmol/L
Free T3	17.1 H	2.8-7.1 pmol/L

Anti-TSH receptor antibodies were not done.

**Figure 1:**
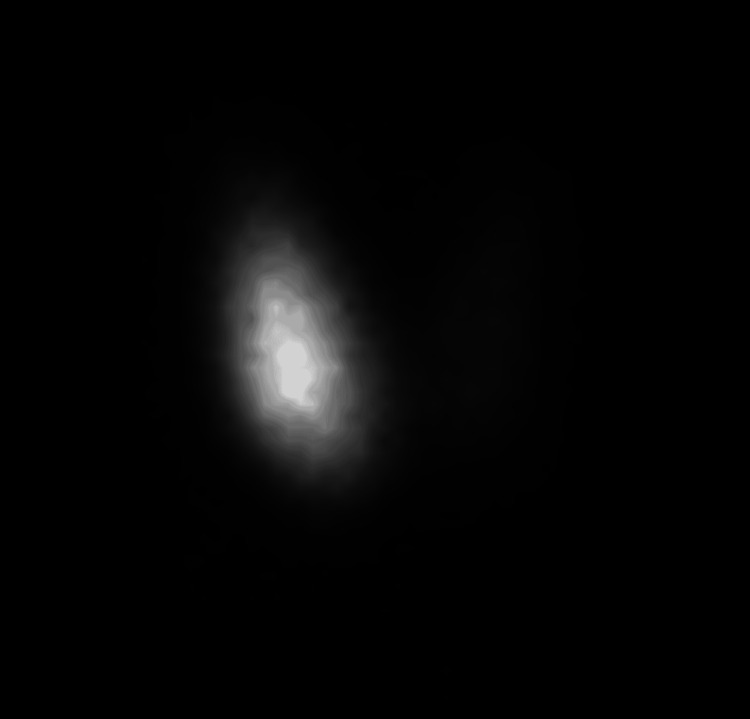
A 20-minute delayed anterior planar image of technetium-99m pertechnetate thyroid scintigraphy demonstrates an asymmetrically enlarged right thyroid lobe with relatively homogeneous dense radiotracer uptake and smooth outline. The left thyroid lobe is normal in size with homogeneous near-total suppression of radiotracer uptake.

Technetium-99m (Tc-99m) thyroid scintigraphy was requested and revealed an asymmetrically enlarged right thyroid lobe with diffuse hyperemia, relatively homogeneous dense radiotracer uptake, and a smooth outline. The left thyroid lobe was normal in size with homogeneous near-total suppression of radiotracer uptake. The background and salivary gland's radiotracer activities were suppressed (Figure 1).

**Figure 2:**
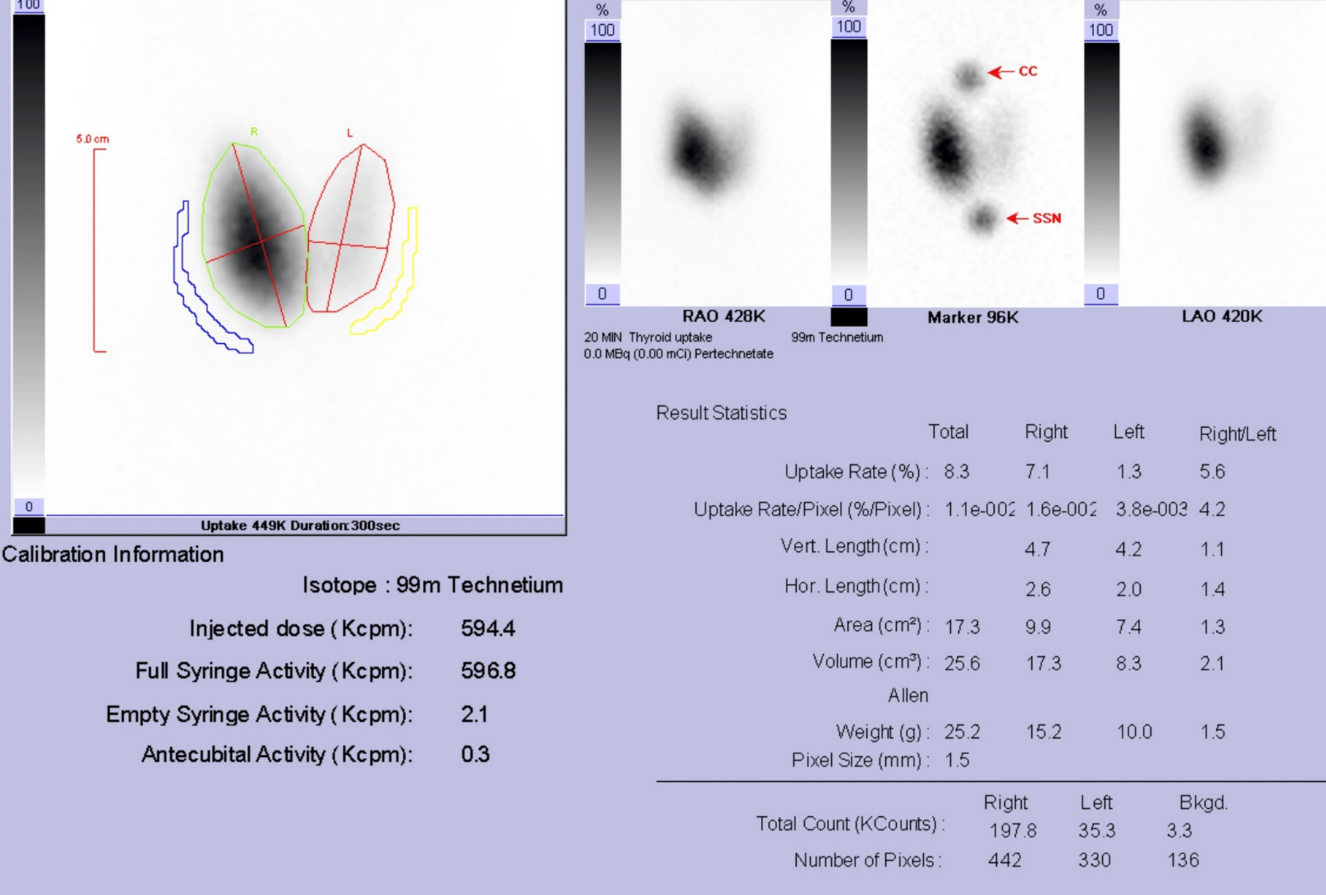
Post-processing automatic calculation of the thyroid uptake value demonstrates asymmetric increased right thyroid lobe uptake value.

The total thyroid uptake was increased and measured 8.3% (normal Tc99m thyroid uptake was 1% to 4%). The differential thyroid uptake value was as follows: right lobe 7.1%, left lobe 1.3% (Figure 2).

**Figure 3:**
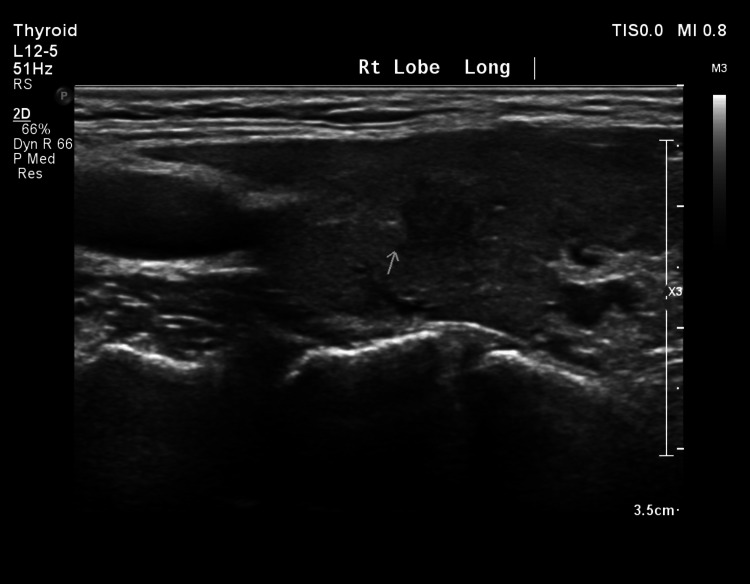
A long-axis ultrasonographic view of the right thyroid lobe demonstrates a central ill-defined hypoechoic area

A complementary thyroid ultrasound was done on the same day to clarify the right thyroid lobe, which revealed an enlarged right thyroid lobe with increased vascularity with a small ill-defined hypoechoic area measuring around 0.6 x 0.5 cm; morphologically based on the American College of Radiology Thyroid Imaging Reporting and Data System (ACR TI-RADS), it had summed points of four to six points rendering it moderately suspicious (Figure 3) [13]. Follow-up was advised due to its small size.

Then, the patient was initiated on anti-thyroid medication (carbimazole 15 mg twice a day). However, the patient and clinician were worried about the thyroid ultrasound findings, and fine needle aspiration (FNA) was requested. 

**Figure 4:**
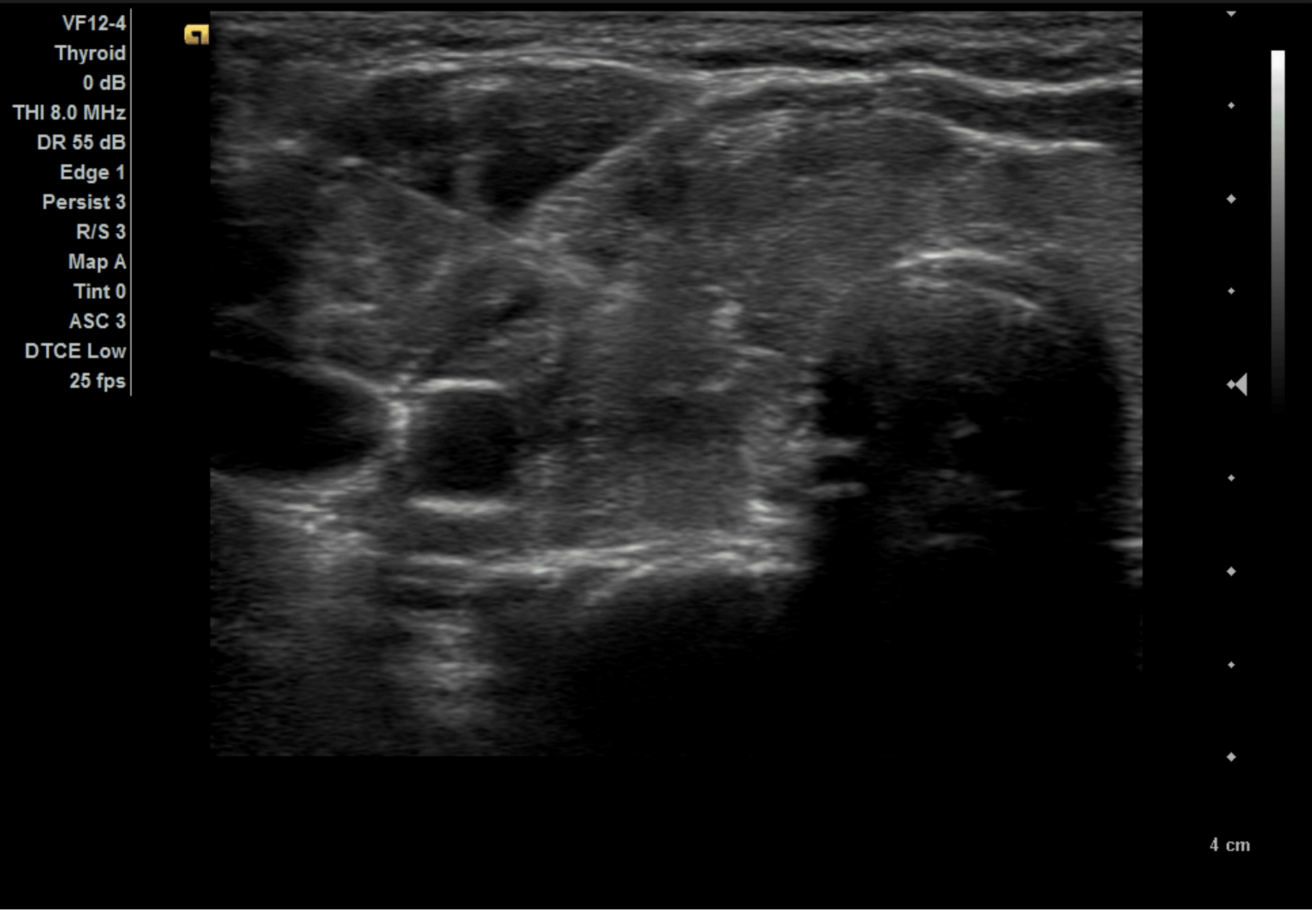
An ultrasonographic view of the right thyroid lobe demonstrates a passed needle through an ill-defined hypoechoic area.

The FNA was done under sterile conditions and ultrasound guidance, and three passes of FNA were made from the ill-defined heterogeneously hypoechoic area within the right thyroid lobe utilizing a 23-gauge needle (Figure 4). The histopathological results showed papillary thyroid carcinoma (PTC).

**Figure 5:**
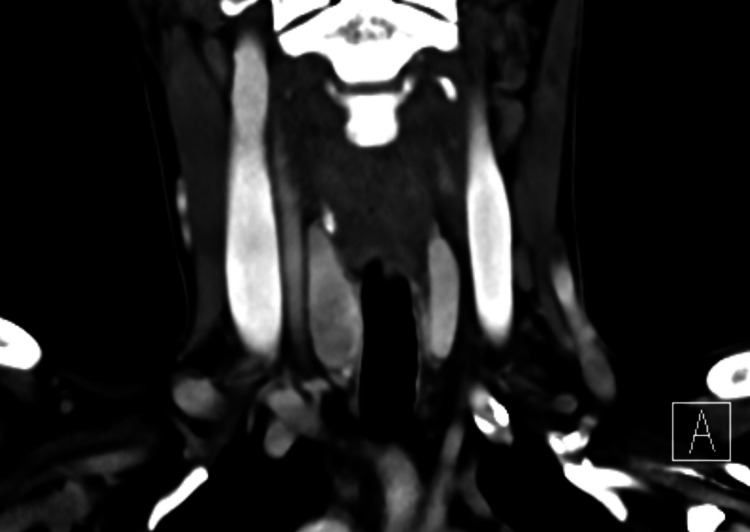
The selected coronal image of the neck demonstrates diffuse right thyroid lobe enlargement with heterogeneously reduced enhancement as compared to the normal contralateral thyroid lobe.

A CT scan of the neck was requested for staging workup, which revealed a slightly heterogeneously hypo-enhancing area in the right thyroid lobe with no definitive nodule based on the CT scan appearance (Figure 5). No suspicious enlarged cervical lymphadenopathy was noted.

**Figure 6:**
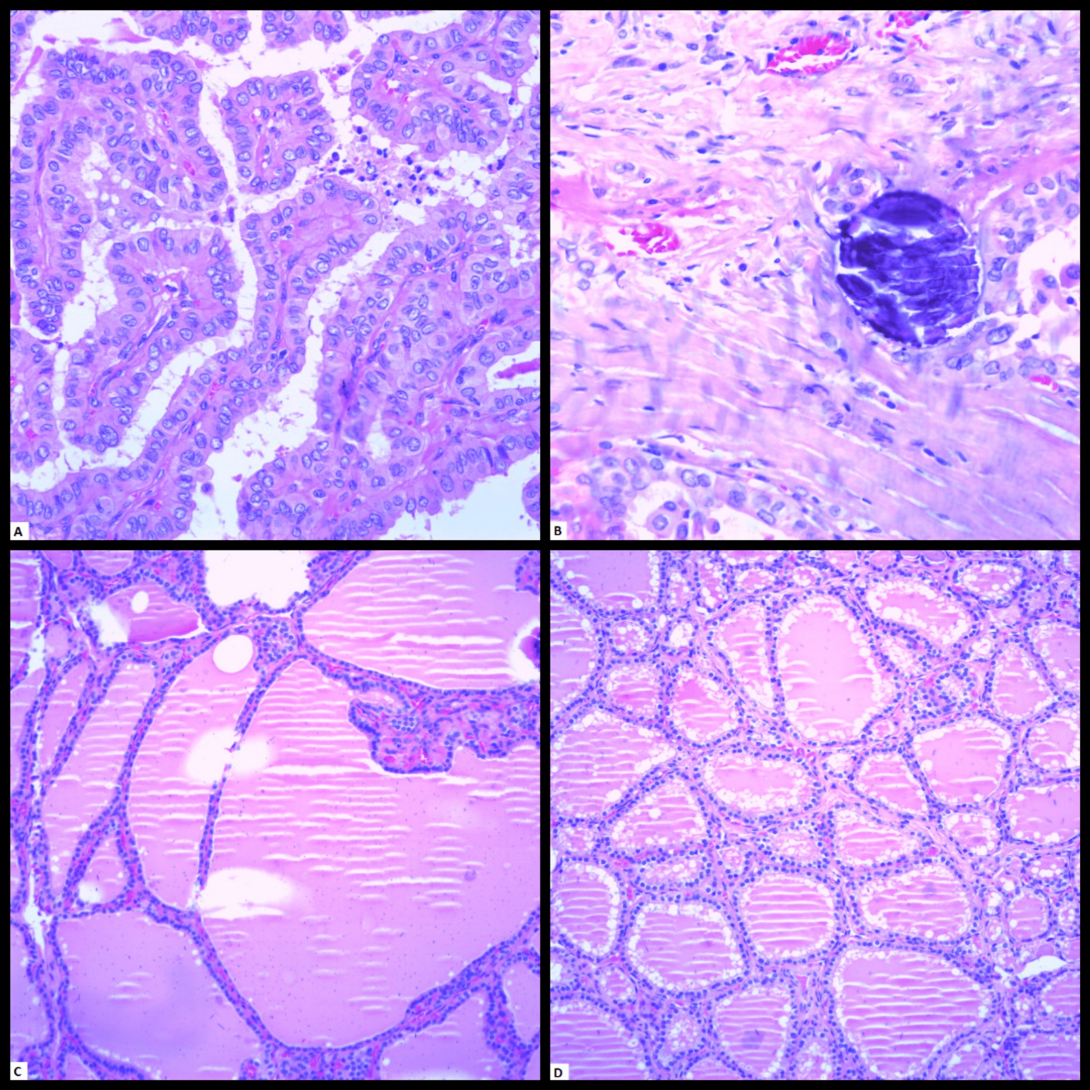
Histology from the total thyroidectomy showing the focus of the papillary thyroid carcinoma A. Fibrovascular cores of papillary thyroid carcinoma demonstrating nuclear enlargement, overlapping, irregular nuclear contour, frequent grooves, chromatin clearing (hematoxylin & eosin stain, 200X magnification). B. Concentric laminated psammomatous calcification (hematoxylin & eosin stain, 200X magnification). C. Features of Graves' disease, including hyperplastic follicles with occasional papillary infoldings into the lumen in the right lobe (hematoxylin & eosin stain, 200X magnification). D. Peripheral scalloping of colloid in the left lobe (hematoxylin & eosin stain, 200X magnification).

Then, the patient underwent total thyroidectomy after controlling the thyroid hormone status. The histopathological result was unifocal right thyroid lobe PTC with tall cell features in the background of bilateral Graves' disease; pathologic stage pT1b (Figures 6A-6D).

The synoptic pathological report is tabulated in Table 3. Currently, the patient is on daily levothyroxine and referred to the oncology center for follow-up and further management.

**Table 3:** The synoptic pathological report of total thyroidectomy

Parameter	Findings
Procedure	Total thyroidectomy
Tumor focality	Unifocal
Tumor site	Right lobe
Tumor size	1.1 cm in greatest dimension
Histologic type	Papillary carcinoma, with tall-cell features
Tumor proliferative activity	Less than 3 mitoses per 2 mm²
Tumor necrosis	Not identified
Angioinvasion	Not identified
Lymphatic invasion	Not identified
Extrathyroidal extension	Not identified
Margin status	All margins negative for carcinoma
Pathologic stage	pT1b

## Discussion

Graves’ disease is the most common cause of hyperthyroidism and is often accompanied by diffuse goiter and thyroid ophthalmopathy [1-2]. Historically, the Merseburg triad has been defined as diffuse thyroid goiter, exophthalmos, and palpitations, common manifestations of Graves’ disease [14]. Females are affected more frequently than males [1-2]. 

Diffuse thyroid goiter is the most common manifestation of Graves’ disease, typically involving both lobes [1-2]. The pathophysiology of Graves’ disease involves a complex interplay of genetic, hormonal, and environmental factors, leading to an autoimmune response against thyroid antigens [2]. However, the precise mechanisms underlying diffuse thyroid enlargement remain unclear. Unilateral thyroid lobe enlargement is a rare manifestation of Graves’ disease [3-5].

Several hypotheses have been proposed to explain unilateral involvement in Graves’ disease. Some suggest that pre-existing structural or functional changes within the thyroid gland may contribute, while others propose a differential expression of antigens between the two thyroid lobes [3]. One hypothesis suggests that the classical diffuse form of Graves' disease can start with unilateral involvement in thyroid scintigraphy presentation and then progress to the diffuse form; this hypothesis is supported by our case and by Sakata et al., who report two cases of unilateral Graves' disease managed by ipsilateral hemithyroidectomy, and then the patients developed hyperfunctioning contralateral lobe [15].

Stimulation of the TSH receptor by anti-TSH receptor antibodies leads to proliferation and increased function of thyroid tissue, resulting in excessive thyroid hormone production and diffuse thyroid enlargement [3]. This stimulation and proliferation of thyroid tissue may also increase the risk of malignancy [3,11]. Since TSH levels are typically very low in Graves’ disease, there is no clear association between TSH levels and thyroid malignancy in affected individuals [3,9-11]. This raises the hypothesis that anti-TSH receptor antibodies may play a role similar to that of TSH in promoting malignancy [3,11]. 

Papillary thyroid carcinoma is the most common type of thyroid malignancy in patients with Graves' disease [8]. A review of the literature suggests that thyroid cancer in Graves' patients tends to present with more aggressive histological features and a higher likelihood of distant metastasis compared to euthyroid patients [3,8].

Clinical evaluation, including physical examination, laboratory findings, dedicated thyroid ultrasound, and thyroid scintigraphy, plays a crucial role in diagnosing, risk stratifying, and monitoring Graves’ disease and its complications. Unilateral thyroid gland enlargement, accompanied by clinical manifestations of hyperthyroidism and uniform unilateral increased thyroid uptake on scintigraphy not explained by a large nodule on ultrasound, is diagnostic for unilateral Graves’ disease, as seen in our case.

Incidentally, thyroid nodules with suspicious ultrasound features, particularly in Graves’ disease patients, may require further assessment via FNA, as was necessary in our case.

## Conclusions

In summary, Graves’ disease is a common autoimmune condition leading to diffuse thyroid enlargement and hyperthyroidism. However, unilateral involvement in Graves’ disease is rare and has unique diagnostic challenges. Our case highlights the importance of considering unilateral Graves’ disease in patients presenting with unilateral thyroid enlargement and hyperthyroidism. The rare occurrence of ipsilateral thyroid malignancy in unilateral Graves’ disease emphasizes the need for a thorough evaluation, including dedicated ultrasound, thyroid scintigraphy, and FNA for suspicious nodules. This case contributes to the limited literature on unilateral Graves’ disease with concurrent thyroid malignancy and represents the first reported case in Saudi Arabia, emphasizing the need for heightened awareness and careful monitoring in similar presentations.
